# Bifunctional Bioactive Polymer Surfaces with Micrometer and Submicrometer-Sized Structure: The Effects of Structure Spacing and Elastic Modulus on Bioactivity

**DOI:** 10.3390/molecules24183371

**Published:** 2019-09-16

**Authors:** Sarah M. Elsayed, Vania Tanda Widyaya, Yasir Shafi, Alice Eickenscheidt, Karen Lienkamp

**Affiliations:** 1 Freiburg Center for Interactive Materials and Bioinspired Technologies (FIT) and Department of Microsystems Engineering (IMTEK), Albert-Ludwigs-Universität, Georges-Köhler-Allee 105, 79110 Freiburg, Germany; 2 Department of Advanced Technology and New Materials Research Institute, City of Scientific Research and Technology Applications, New Borg El-Arab City, Alexandria 21934, Egypt

**Keywords:** chemical surface modification, colloidal lithography, microcontact printing, structured polymer surfaces, surface-cell interactions

## Abstract

This study presents a comparison of two types of bifunctional structured surface that were made from the same polymer—an antimicrobial polycation (a synthetic mimic of an antimicrobial peptide, SMAMP) and a protein-repellent polyzwitterion (poly(sulfobetaines), PSB). The first type of bifunctional surface was fabricated by a colloidal lithography (CL) based process where the two polymers were immobilized sequentially onto pre-structured surfaces with a chemical contrast (gold on silicon). This enabled site-selective covalent attachment. The CL materials had a spacing ranging from 200 nm to 2 µm. The second type of structured surface (spacing: 1–8.5 µm) was fabricated using a microcontact printing (µCP) process where SMAMP patches were printed onto a PSB network, so that 3D surface features were obtained. The thus obtained materials were studied by quantitative nanomechanical measurements using atomic force microscopy (QNM-AFM). The different architectures led to different local elastic moduli at the polymer-air interface, where the CL surfaces were much stiffer (Derjaguin-Muller-Toporov (DMT) modulus = 20 ± 0.8 GPa) compared to the structured 3D networks obtained by µCP (DMT modulus = 42 ± 1.1 MPa). The effects of the surface topology and stiffness on the antimicrobial activity against *Escherichia coli*, the protein repellency (using fibrinogen), and the compatibility with human gingival mucosal keratinocytes were investigated. The softer 3D µCP surfaces had simultaneous antimicrobial activity, protein repellency, and cell compatibility at all spacings. For the stiffer CL surfaces, quantitative simultaneous antimicrobial activity and protein repellency was not obtained. However, the cell compatibility could be maintained at all spacings. The optimum spacing for the CL materials was in the range of 500 nm–1 µm, with significantly reduced antimicrobial activity at 2 µm spacing. Thus, the soft polymer network obtained by µCP could be more easily optimized than the stiff CL surface, and had a broader topology range of optimal or near-optimal bioactivity.

## 1. Introduction

Biofilm formation on medical devices such as catheters or implants is a critical problem in modern healthcare, leading to severe infections of a large number of patients worldwide every year [1,2,3]. Biofilms are defined as matrix-enclosed bacterial aggregates attached to a surface, in which the constituent bacteria have a different activity and metabolism than their planktonic counterparts [4,5]. Due to the extracellular matrix which protects the bacteria inside the biofilm, it is difficult to eradicate biofilm bacteria. This may require a 100−1000 times higher dose of antibiotics compared to planktonic bacteria of the same strain [3]. This problem is getting even worse when antibiotic-resistant bacterial strains are involved [6,7]. Therefore, it is essential to inhibit biofilms on biomaterials by slowing down their initial steps of formation.

Several methods and techniques have been recently reported to fight biofilm formation, e.g., suppressing bacterial adhesion, or functionalizing surfaces with antimicrobially active substances. One important technique in this context is chemical surface modification with antimicrobial polycationic polymer coatings, or with polyzwitterion-based protein-repellent polymers. Cationic antimicrobial coatings can kill bacteria by interacting with their negatively charged membranes. However, when cationic surfaces thus “capture” negatively charged bacteria and other biomolecules, this causes contamination and deactivation of the surface and can initiate biofilm formation [8]. This problem is the weak point of such surfaces, especially when they are exposed to large amounts of bacteria. Polyzwitterions, on the other hand, have an equal number of positive and negative charges and are strongly hydrophilic. They can bind significant amounts of water and do not disturb the hydrogen bond network of the surrounding fluid. Thus, adhesion of proteins and bacteria on these surfaces does not result in an overall gain of free energy for the system, and the surfaces are therefore protein-repellent [9,10,11,12,13]. However, protein-repellent polymer coatings are vulnerable to contamination by other entities, e.g., lipids. Lipid adhesion to such a surface may facilitate settling and proliferation of single bacteria on protein-repellent surfaces. This one pathogen can then form a biofilm in less than 24 h [14]. Thus, chemical surface modification strategies with one “line of defense” against bacteria are beneficial to prevent biofilm formation for short times, but cannot prevent biofilm formation in long term applications.

The high demand for longer lasting anti-biofilm activity has motivated scientists to develop bifunctional materials with combined antimicrobial activity and protein repellency [8,15,16,17,18]. The idea of this concept is that protein- and bacteria-repellent “walls” and antimicrobial “knights” incorporated into the same material could slow down the initial stages of biofilm formation more efficiently than monofunctional materials (“It takes walls and knights to defend a castle”) [19]. Such bifunctional materials can be synthesized, for example, by embedding leachable antimicrobial agents, such as nanoparticles, antibiotics or other biocides into protein-repellent polymer matrices [20,21], or by hydrolytically releasing covalently bound antimicrobial agents from a protein-repellent material [22,23,24,25]. Another interesting design involves switching the surface charge of the materials from cationic/contact killing to zwitterionic/protein-repellent [25,26]. Other surfaces with switchable bioactivities triggered by temperature changes have also been reported [27,28]. Even though these concepts are highly attractive from the academic perspective, they are difficult to implement in real-life applications or clinical settings.

Two other critical materials properties that affect biofilm formation are the stiffness and the topology of a surface [29,30,31,32,33]. For example, it was recently found that the motility of *Escherichia coli* bacteria on stiff cross-linked poly(dimethylsiloxane) (PDMS) was higher than on soft PDMS (surface stiffness: 2.6 to 0.1 MPa), i.e., bacteria attached less firmly to the stiffer surfaces [33]. It was also found that the attached bacteria were more elongated and more sensitive to antibiotics on the softer surfaces [34]. However, differing results were also reported: fewer *E. coli* and *Staphylococcus aureus* bacteria adhered to soft hydrogels made from poly(ethylene glycol) dimethacrylate (PEGDA, Young’s modulus: 44 to 6500 kPa) [35]. Similarly, *Staphylococcus epidermis* adhesion to polyelectrolyte multilayer thin films made from poly(allylamine) hydrochloride and poly(acrylic acid) decreased on the softer surfaces (Young’s modulus: 0.8–80 MPa) [36]. Overall, the growing evidence seems to suggest that the effect of surface stiffness on bacterial adhesion depends on the hydrophobicity of the surface [37]. Bacterial attachment to hydrophobic surfaces seems to increase for softer materials, and to decrease on softer hydrophilic surfaces [37]. This is evidence that bacteria can sense the mechanical properties of a surface, and it is supposed that bacteria have specific genes that are expressed in response to material stiffness [33,34,35,36,37].

Furthermore, surface topography influences bacterial adhesion through the size, shape, and orientation of the surface features [38,39]. For instance, features larger than the bacterial cells deter proliferation [32,39]; equally or smaller sized features affect bacterial orientation and thereby reduce adhesion [40,41]. Nano-sized surface features with high aspect ratios were even intrinsically bactericidal because the high curvature of the tips ruptured the bacterial membranes [42,43,44].

Recently, surface structuring was combined with bifunctional chemical surface modification. For example, microstructured bifunctional surfaces made from contact-killing polycations and protein-repellent polyzwitterions were reported. A checkered material consisting of 4 μm^2^ squares made from poly(quaternary ammonium salt) and poly(sulfobetaine) brushes reduced the adhesion of *E*. *coli* by 70−93% compared to an untreated polyamide substrate [45]. We have previously reported bifunctional micro- and submicrometer-structured polymer surfaces made from polyoxanorbornene-based cationic synthetic mimics of antimicrobial peptides (SMAMPs) and zwitterionic poly(sulfobetaines) (PSB). Their sub-micrometer and micrometer sized surface features were obtained by colloidal lithography (CL) and microcontact printing (µCP). Surfaces obtained by CL [46] were structured surface-attached polymer monolayers with spacings ranging from 200 nm to 1 μm. Biological studies showed that the bifunctional surfaces with 1 μm spacing were fully antimicrobially active against *E. coli* and strongly fibrinogen-repellent. At smaller spacings, a reduced antimicrobial activity but an enhanced fibrinogen-repellency was observed [46]. The surfaces obtained by µCP were 3D structured surface-attached polymer hydrogels with spacings ranging from 1 to 8.5 μm [47]. Biological studies showed that the bifunctional surfaces with 1 and 2 μm spacing were fully antimicrobially active against *E. coli* and *S. aureus*, fully fibrinogen-repellent, and nontoxic to human gingival mucosal keratinocytes [47]. However, at 8.5 μm spacing, the antimicrobial activity against *S*. *aureus* was slightly decreased [47]. These results show that the upper size limit for full antimicrobial activity of these patterned polymer hydrogels is between 2 and 8.5 μm spacing [47]. From here arises the question: what is the upper limit of the dual activity of the (much stiffer) surface-attached polymer layers obtained by colloidal lithography? Is it the same, or different from the µCP upper limit? And if it is different, how do other parameters, particular the surface stiffness, affect protein adhesion and antimicrobial activity? Answering these questions is the target of the work presented here.

## 2. Results

### 2.1. Study Design

**Figure 1:**
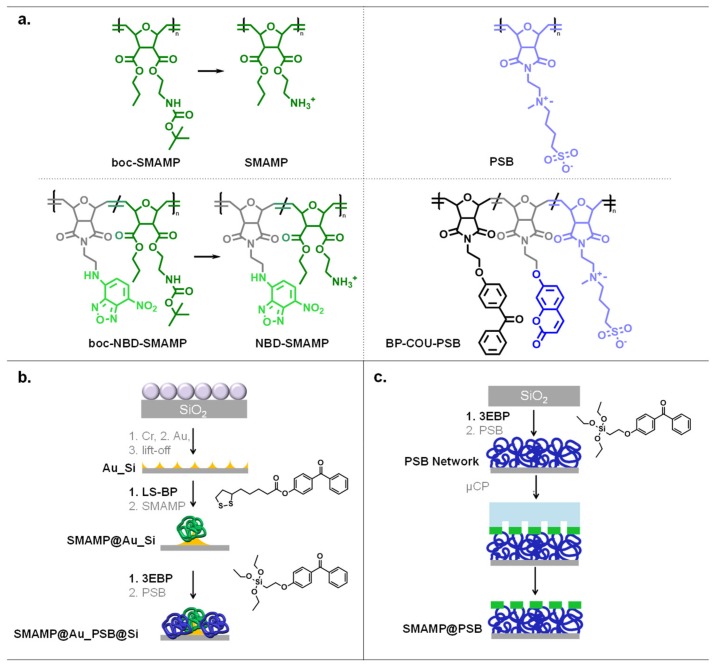
(**a**) Polymers used: **boc-SMAMP** and **PSB** for CL; **boc-NBD-SMAMP** and **BP-COU-PSB** for µCP. The boc protective groups were removed after the structuring process to obtain active SMAMP groups. (**b**) CL fabrication process. A monolayer was formed from polystyrene colloids (2 µm) on a silicon wafer and used as a lithographic mask, through which chromium and gold were evaporated. After mask removal, the gold islands (yellow) were functionalized with lipoic acid disulfide benzophenone (**LS-BP**) and **boc-SMAMP** (green). Treatment with HCl activated the SMAMP. The silicon background was functionalized with triethoxy benzophenone silane (**3EBP**) **PSB** (blue). (**c**) µCP fabrication process: A silicon wafer was functionalized with **3EBP** followed by immobilization of the **PSB** network (blue, made from **BP-COU-PSB**). **boc-NBD-SMAMP** (green) was printed onto the network using a PDMS stamp (light blue). The printed **boc-NBD-SMAMP** pattern was surface-attached by UV-irradiation (λ = 254 nm, energy = 3 J cm^−2^), giving **SMAMP@PSB** after removal of the boc protective groups with HCl.

The aim of this study was to compare the physical and biological properties of bifunctional polymer-functionalized surfaces. Two types of materials were studies: structured polymer monolayers obtained by colloidal lithography (CL), and 3D-structured polymer networks fabricated by microcontact printing (µCP). They were each made from two components: an antimicrobial polycationic synthetic mimic of an antimicrobial peptide (**SMAMP**), and the protein-repellent polyzwitterion poly(sulfobetaine) (**PSB**, Figure 1a). Both types the CL and µCP materials consisted of periodic polymer patches with similar spacings. Thus, when immersed into aqueous medium, the functional groups at the polymer-liquid interface would be the same, yet their distribution would be different. Thus, a direct comparison of these materials and their structure-property relationships should lead to a clearer picture of the effect of polymer surface architecture on antimicrobial activity and protein repellency. In particular, the structured networks were significantly thicker and had lower local elastic moduli near the polymer-liquid interface than the structured monolayers. This would allow to assess the effect of the elastic modulus on the surface bioactivity. Homogeneous **SMAMP** and **PSB** monolayers and networks [46,47] were added as reference surfaces to the study.

The CL and µCP process to generate these structured materials has been reported previously [46,47]. We here report additional data to complete the series of CL materials (2 µm spacing). Additionally, to assess the effect of surface architecture on the mechanical properties of the surface, representative samples were studied by Quantitative Nanomechanical Atomic Force Microscopy—(QNM-AFM). This data was compared to the additional and previously reported physical and bioactivity data of the CL and µCP materials series to gain new insights about the effects of spacing and mechanical properties of these bifunctional materials on their bioactivity.

### 2.2. Surface Fabrication

#### 2.2.1. Colloidal Lithography

Bifunctional 2D structured polymer monolayers were made from the antimicrobial polymer **SMAMP** and the protein-repellent **PSB** (Figure 1a). The underlying surface pattern was obtained by colloidal lithography (CL), as reported previously [46]. The method is illustrated in Figure 1b. Briefly, first a monolayer of close-packed polystyrene colloids was assembled to a monolayer and used as a lithographic mask. By evaporating a thin layer of chromium (about 5 nm, adhesive layer) followed by a layer of gold (50 nm) through the colloids, and then removing the colloid mask, a surface with a chemical contrast (gold on silicon, **Au_Si**, Figure 1b) was obtained [46]. The gold islands were then reacted with the gold-selective anchor group **LS-BP** (Figure 1b), and the **boc-SMAMP** polymer (Figure 1a) was spin coated onto the surface. UV-irradiation at λ = 254 nm triggered a C,H-insertion crosslinking reaction between the aliphatic C-H group of the polymer and the benzophenone moietly of **LS-BP**. Thus, **boc-SMAMP**-functionalized gold islands on a silicon background were obtained (**SMAMP@Au_Si**, Figure 1b). The silicon patches were reacted with the silicon-selective **3EBP** anchor group (Figure 1b), and the **PSB** polymer was applied by spin-coating. It was surface-attached through the benzophenone moiety of **3EBP** by UV irradiation. To activate the antimicrobial functionality of the material, the **boc** protective group of the **boc-SMAMP** patches were removed by treatment with HCl, yielding **SMAMP@Au_PSB@Si** (Figure 1b). Bifunctional CL materials with a lateral spacing of 1 µm, 500 nm and 200 nm have been previously reported [46]. To complete the series, materials with 2 µm spacing were obtained, and are reported below.

#### 2.2.2. Microcontact Printing

Bifunctional 3D surface-attached polymer networks were obtained by microcontact printing (µCP, Figure 1c) as reported previously [47,48,49]. They were made from **BP-COU-PSB** and **boc-NBD-SMAMP**. Both of these polymers were similar to the ones used for CL; the only difference was that the **boc-NBD-SMAMP** contained a small fraction of repeat units that carried the fluorophore nitrobenzoxadiazole (NBD) in addition to the boc-SMAMP repeat units. The **BP-COU-PSB** contained a small fraction of repeat units that carried both the UV-active benzophenone cross-linker and a coumarin fluorophore in addition to the protein-repellent PSB repeat units. These polymers were described previously [47,49]. The benzophenone repeat units of **BP-COU-PSB** were used to cross-link the polymer by UV-irradiation, so that a thick crosslinked network was formed. The NBD moeities also act as UV crosslinkers and yield structured 3D polymer patches upon UV irradiation. The µCP materials were prepared by first surface functionalizing a silicon substrate with the **3EBP** anchor group (Figure 1c). **BP-COU-PSB** solution was then spin-coated onto the **3EBP**-functionalized surface, followed by UV-irradiation. This gave a surface-attached **BP-COU-PSB** network. A **boc-NBD-SMAMP** pattern was then printed onto the network surface using a PDMS stamp. The PDMS stamp had parabolic microstructures with a spacing of 1 µm, 2 µm, or 8.5 µm as reported previously [47,49], and was inked with **boc-NBD-SMAMP** solution. The stamp was then lifted off, and the printed **boc-NBD-SMAMP** pattern was UV-irradiated to give 3D surface-attached **boc-NBD-SMAMP** patches. They were then treated with HCl to remove the **boc** protective group, yielding the bifunctional microstructured **SMAMP@PSB** surfaces (Figure 1c).

### 2.3. Physical Surface Characterization

#### 2.3.1. Atomic Force Microscopy and Contact Angle Measurements

**Figure 2:**
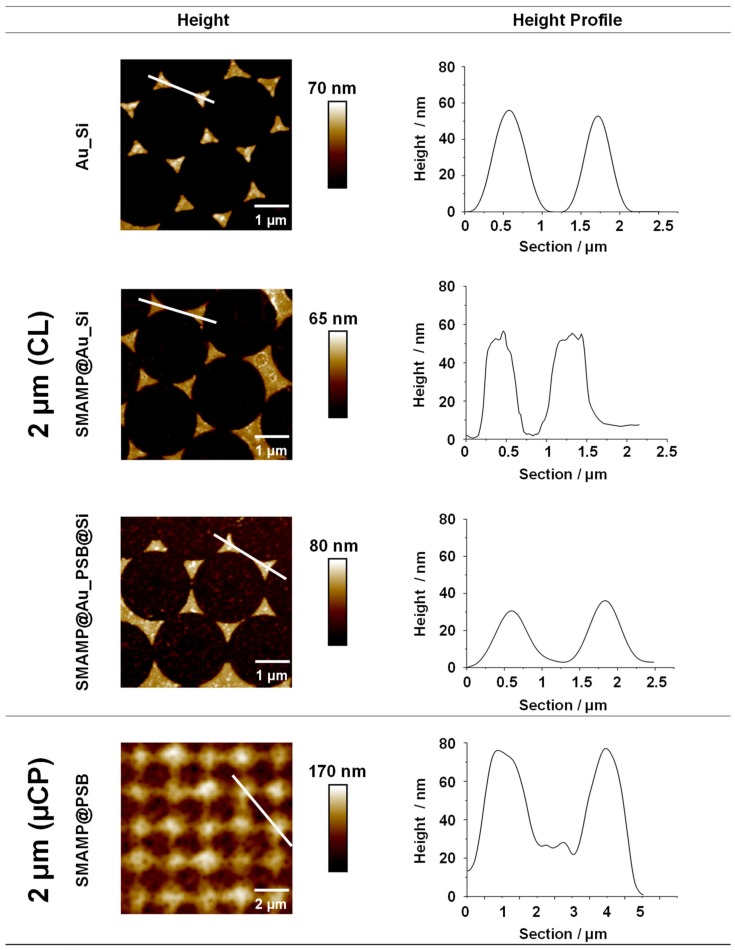
AFM height images and height profiles of 2D- and 3D structured surfaces with spacing of 2 µm. The 2D structured surfaces (**Au_Si**, **SMAMP@Au_Si**, and **SMAMP@Au_PSB@Si**) were fabricated using CL, and the 3D structured surface (**SMAMP@PSB**) was fabricated using µCP. The line (white) in the height images indicates the position where the height profiles were taken.

The CL and µCP structures were characterized by atomic force microscopy (AFM) after each processing step. Height images and height profiles for the CL and µCP structures with 2 µm spacing are shown in Figure 2. Further data can be found in Figure S1 (height image of the 2 µm lithographic mask) and for the other spacings in our previous publications [46,47].

The height images and profiles in Figure 2 show the products of the CL process after each processing step. The gold islands in **Au_Si** were well-defined triangles with a mean height of about 55 nm and smooth height profiles, while the **boc-SMAMP**-functionalized gold islands of **SMAMP@Au_Si** had slightly blurred domain edges and slightly irregular height profiles due to polymer binding. This is consistent with previous reports [46,48]. The height difference between the Si background and the Au islands is still about 55 nm, which confirms that only a monolayer of **boc-SMAMP** was immobilized onto the gold islands. (As already reported previously, the thickness of a **SMAMP** monolayer is only about 2 nm [49]). The bifunctional **SMAMP@Au_PSB@Si** surfaces again have smoother domain edges as a polymer layer now covers the entire material. The PSB-covered patches show a granular morphology, which is characteristic for polyzwitterionic polymer layers [50]. Notably, the distance in z-direction between Au and Si domains was reduced to 35 nm, which emphasizes that more than a monolayer of **PSB** was immobilized on Si background, possibly a result of additional ionic network formation of the polyzwitterionic **PSB** polymer. Thus, immobilization of both polymers on their respective sites was achieved. This was further supported by the second characterization method, contact angle measurements, Table S1. The results show that the partially functionalized surfaces (**SMAMP@Au_Si**, ***θ_receding_*** = 48°) were more hydrophobic than the surfaces additionally covered with the hydrophilic **PSB** (**SMAMP@Au_PSB@Si**, ***θ_receding_*** = 36°). This also matches our previously reported results [46]. In order to determine the average thickness after each fabrication step, surface plasmon resonance (SPR) reflectivity curves were recorded. After each processing step, a shift of the reflectivity minimum to higher angles (Figure S2) indicated an increase in the average layer thickness. The thickness of each layer was determined by modelling the reflectivity curves with the Fresnel equations; the results are listed in Table S2.

A representative AFM height image and profile of the bifunctional material fabricated by µCP with 2 µm spacing is also shown in Figure 2. The data shows that the structured surface had a granular blurred morphology. This granular morphology comes from the underlying **PSB network**, as previously reported [47]. The thickness of the **PSB network** was about 80 nm according to SPR measurements [47]. The height of the printed antimicrobial **SMAMP** microstructures was also about 80 ± 20 nm, as determined by AFM. This is siginicantly thicker than a polymer monolayer, indicating that a network formed by UV-induced cross-linking of the NBD units. Thus, it was confirmed by AFM that the bifunctional µCP structures had an all-polymer 3D architecture. SPR data for this sample has been reported previously, together with the data for µCP architectures with 1 µm and 8.5 µm spacing [47].

**Figure 3:**
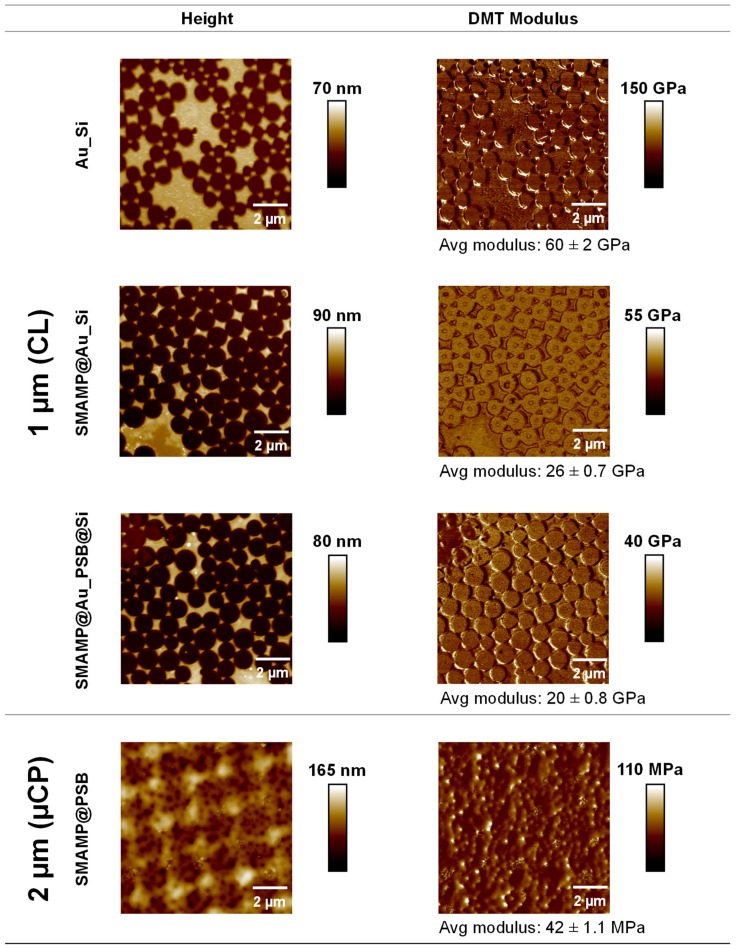
AFM height and QNM images of the 2D structured surfaces fabricated by CL (top rows, 1 µm spacing), and 3D structured surfaces obtained by µCP (bottom row, 2 µm spacing). Avg = average.

The mechanical properties of selected CL and µCP surfaces were analyzed with Quantitative Nanomechanical AFM (QNM-AFM, Figure 3). The Derjaguin-Muller-Toporov (DMT) model was used. We here report the DMT modulus $E^{*}$, which is related to the Young’s modulus of the sample ($E_{s}$) by

(1) $$E^{*}={\left[\frac{1-v_{t}{}^{2}}{E_{t}}+\frac{1-v_{s}{}^{2}}{E_{s}}\right]}^{-1}\approx \frac{E_{s}}{1-v_{s}{}^{2}}$$

where $E_{t}$ is the Young’s modulus of the AFM tip, and $v_{t}$ and $v_{s}\;$ are the Poisson ratios of tip and sample, respectively. $E^{*}$ was reported because we were interested in modulus differences rather than absolute numbers, and wanted to avoid making assumptions about the magnitude of the Poisson ratios. (In any case, the numerical difference to $E_{s}$ would be small, as $v_{s}$ is between 0.3 to 0.5 for samples with an $E_{s}$ less than 10 GPa.)

The bifunctional substrate **Au_Si** (spacing 1 µm) was a stiff material with a DMT modulus of 60 ± 2 GPa. For the **SMAMP@Au_Si** materials, the stiffness at the surfaces reduced to 26 ± 0.7 GPa, confirming the presence of the significantly softer **SMAMP** on the surfaces. The **SMAMP@Au_PSB@Si** surfaces had a stiffness of 20 ± 0.8 GPa, i.e., they had a modulus on the same order of magnitude as expected. From the relative small modulus difference between the bare **Au_Si** sample and the polymer-covered domains, it can be concluded the AFM cantilever could still sense the stiffness of the underlying **Au_Si**, resulting in an overall high local elastic modulus of these thin polymer layers. This is to be expected, as the polymer layer thickness was on the same order of magnitude as the indentation depth. Note that for the QNM measurements of this sample, a defect-rich area was chosen so that the modulus on the islands could be determined more easily and with out artifacts from structure edges. In the case of the bifunctional structured µCL surfaces (**SMAMP@PSB**, spacing 2 µm), the stiffness was significantly decreased to 42 ± 1.1 MPa (Figure 3, respectively). There was no modulus difference between the printed **SMAMP** region and the **PSB** region. This is because the thickness of **PSB** was already significant. Thus, the QNM-AFM measurements confirmed the postulated architectural differences between the CL surfaces, which consisted of thin polymer layers, and the structured surfaces fabricated by µCP, which were sufficiently thick to claim that they are indeed 3D structures with different mechanical properties than the thin 2D layers.

#### 2.3.2. Protein Adhesion Studies

**Figure 4:**
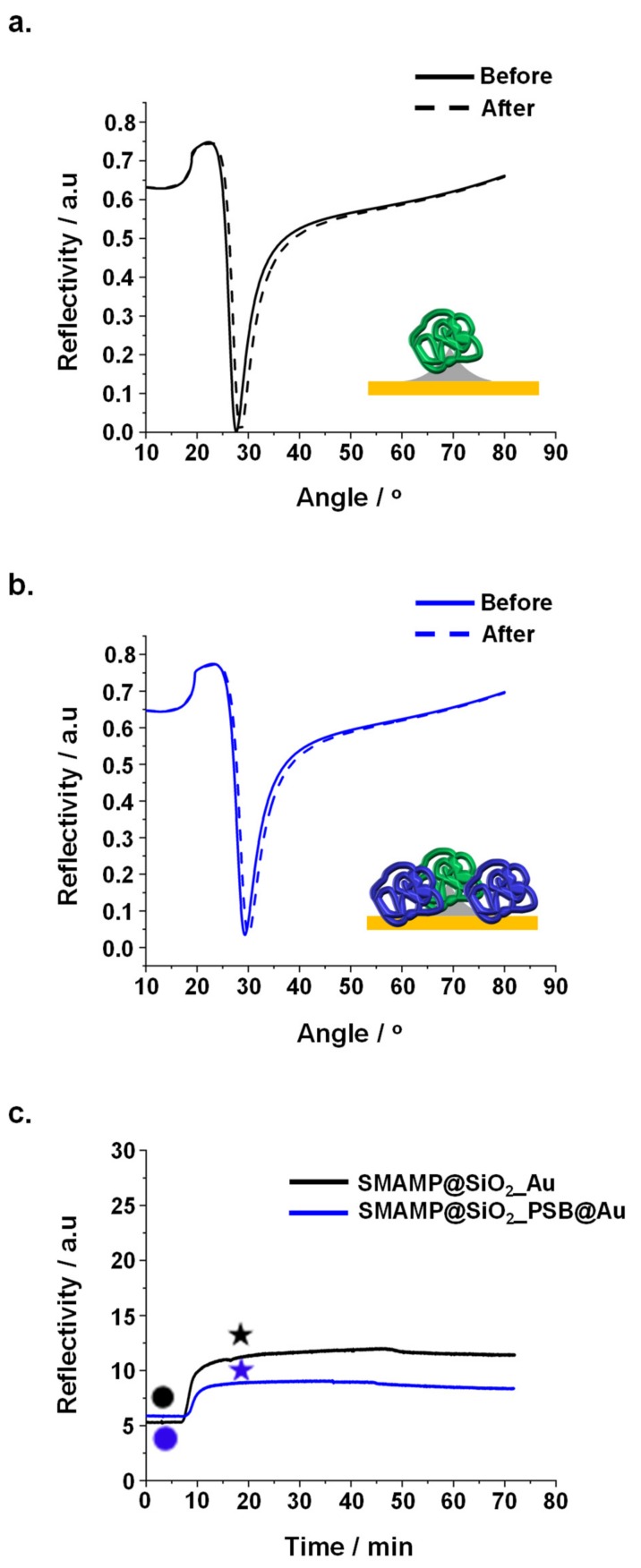
Protein adhesion on surfaces with 2 µm spacing studied by SPR: reflectivity curves of protein adhesion on (**a**). **SMAMP@SiO_2__Au** and (**b**). **SMAMP@SiO_2__PSB@Au**. Angular scans before and after exposure to fibrinogen were recorded (solid curves: dry thickness before fibrinogen adsorption, dashed curve: dry thickness after fibrinogen adsorption). (**c**). SPR kinetics curves of fibrinogen adhesion on **SMAMP@SiO_2__Au** and **SMAMP@SiO_2__PSB@Au**, both with 2 µm spacing. Circles: time points of protein injection; stars: time points of buffer injection.

Fibrinogen adhesion on the CL and µCP samples was studied by surface plasmon resonance spectroscopy (SPR) as described previously [46,47]. Briefly, CL samples with inverse contrast were created by evaporating silicon oxide onto the SPR gold sensor through the colloidal mask, yielding **SiO_2__Au**. The silicon islands of these samples were then functionalized with **3EBP** and **boc-SMAMP** polymer, yielding **SMAMP@SiO_2__Au**. The gold patches were reacted with **LS-BP** and **PSB**, yielding **SMAMP@SiO_2__PSB@Au** after treatment with HCl to remove the **boc** groups. Full angular reflectivity scans were recorded for the dry **SMAMP@SiO_2__Au** and **SMAMP@SiO_2__PSB@Au** samples before and after protein exposure (Figure 4a,b, respectively). The kinetics of the protein adhesion process is shown in Figure 4c. Both procedures have been reported in more detail previously [46,48,50,51]. The adhered protein amount was quantified by fitting the reflectivity curves with the Fresnel equations as described in the Experimental. From the adlayer thickness before and after the protein adhesion, the amount of protein adhered on the surfaces was quantified as mass per area (mA^−1^) using ρ = m V^−1^ and V = t A, where ρ = protein density, m = protein mass, V = protein volume, t = thickness, A = surface area and ρ_fibrinogen_ = 1.085 g cm^−3^ [52]. The data thus obtained for **SMAMP@SiO_2__Au** and bifunctional **SMAMP@SiO_2__PSB@Au** surfaces with spacings from 200 nm to 2 μm are summarized in Table 1, together with the likewise obtained data for µCP samples with 1 – 8.5 µm spacing and unstructured PSB and SMAMP monolayers and networks as reference samples, respectively. The SPR results show that the partially covered **SMAMP@Au_Si** surfaces had high protein adhesion at all spacings (2.8–16.3 ng mm^−2^, respectively), although a clear trend between spacing and protein load could not be observed. For the bifunctional **SMAMP@SiO_2__PSB@Au** surfaces, protein adhesion increased with increasing spacing: while quantitative protein repellency was observed at 200–500 nm spacing, at larger spacings (1–2 µm), 0.2–0.5 ng mm^−2^ of adhered protein was measured. This was substantially lower than the data observed for **SMAMP** (13 ng mm^−2^) and **PSB monolayers** (11 ng mm^−2^). The µCP samples made from polymer networks show a better protein repellency, with protein adhesion of 0 ng mm^−2^ for all spacings. A significant decrease in protein adhesion was also observed for **SMAMP network** (6.6 ng mm^−2^) and **PSB network** (0 ng mm^−2^) compared to the respective monolayers. Thus, it can be concluded that surfaces made from softer polymer networks have a better protein repellency compared to the stiffer polymer monolayers (Table 1).

### 2.4. Biological Surface Characterization

#### 2.4.1. Antimicrobial Assay

**Figure 5:**
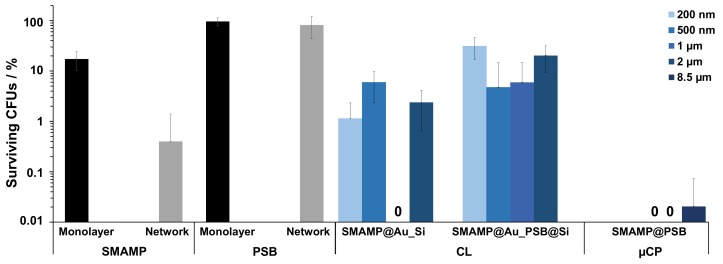
Antimicrobial activity of the different functionalized surfaces against *E*. *coli* bacteria. The normalized percentage of surviving bacteria was plotted for unstructured control surfaces (**SMAMP monolayer** and **network**; **PSB monolayer** and **network**), structured CL surfaces (**SMAMP@Au_Si** and **SMAMP@Au_PSB@Si** with 200 nm, 500 nm, 1 µm and 2 µm spacing, respectively), and structured µCP surfaces (**SMAMP@PSB** with 1 µm, 2 µm, and 8.5 µm spacing, respectively). The data for the monolayer controls and the CL samples with 200 nm, 500 nm and 1 µm spacing are from [46]. The data of the network controls and the structured µCP surfaces are from [47]. The error bars are the standard deviation calculated from at least two independent experiments with five replicates each.

A standardized antimicrobial assay was used to quantify the antimicrobial properties of the above-fabricated surfaces against *Escherichia coli* bacteria, as described before [53]. Shortly, a suspension of *E. coli* (10^6^ bacteria mL^−1^) was sprayed to the samples and incubated for 4 h, then the surviving bacteria (colony forming units, CFUs) were plated out, incubated, and counted. The percentage of CFUs was normalized to the respective growth controls (uncoated silicon wafer), so that the relative antimicrobial activity of the materials could be compared. The data thus obtained for four unstructured control surfaces (**SMAMP** and **PSB** monolayer, **SMAMP** and **PSB** network), the structured CL materials (**SMAMP@Au_Si** and **SMAMP@Au_PSB@Si**), and the µCP materials (**SMAMP@PSB**) is plotted in Figure 5. The **SMAMP monolayer** control surface had a lower antimicrobial activity (17% CFUs) compared to the **SMAMP network** surface (0.4% CFUs). On the other hand, both **PSB** control surfaces were not active with 97% CFUs for the **PSB monolayer** and 82% CFUs for the **PSB network**. For the **SMAMP@Au_Si** samples, a substantial antimicrobial activity was observed, with a quantitative killing of bacteria (0% CFUs) for the 1 µm spacing. At the other spacings, the activity was also high but not quantitative (2% CFUs for 2 µm, 5% CFUs for 500 nm spacing, and 1% CFUs for 200 nm spacing), and no clear trend was observed. Yet these surfaces were still more active than the **SMAMP monolayer**, which had 17% CFUs in this data set. The antimicrobial activity of the bifunctional **SMAMP@Au_PSB@Si** surfaces was non-zero for all spacings and showed an interesting trend. There were 25% CFUs for 200 nm spacing, 4% CFUs for the 500 nm samples, 5% CFUS for the 1 µm spacing, and 17% CFUs for the 2 µm spacing. Thus, there is a maximum activity for the intermediate spacings. The bifunctional **SMAMP@PSB** surfaces obtained by µCP, on the other hand, were highly active against *E. coli* with quantitative or near-quantitative killing for all spacings. From these results, it could be concluded that softer surfaces made from polymer networks had a better antimicrobial activity compared to the stiffer surfaces made from a polymer monolayer.

#### 2.4.2. Cell Compatibility

**Figure 6:**
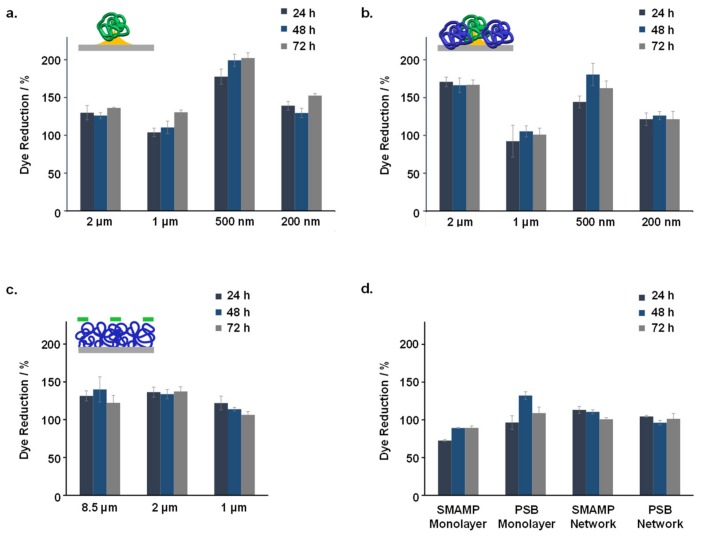
Normalized relative dye reduction (in %) due to keratinocytes grown on the structured and functionalized samples: (**a**). **SMAMP@Au_Si** with 200 nm to 2 µm spacing; (**b**). **SMAMP@Au_PSB@Si** with 200 nm to 2 µm spacing; (**c**). **SMAMP@PSB** samples with 1 µm to 8.5 µm spacing; (**d**). **SMAMP monolayer** and **PSB monolayer**, and **SMAMP network** and **PSB network**. The data for each sample was recorded after 24, 48, and 72 h of incubation time, respectively. The error bars are the standard deviation calculated from two independent experiments using three replicates each.

Three different methods were used to test the compatibility of the structured surfaces with human gingival mucosal keratinocyte (GM-K) cells. Optical microscopy (phase contrast) was used to examine the morphology and density of keratinocytes grown on the fabricated surfaces. A live-dead assay for mammalian cells was used to visualize the ratio of healthy and membrane-compromised cells. Finally, the Alamar Blue assay was performed to quantify the metabolic activity of the GM-K cells, where the reduction of the resazurin dye indicates cell activity. The dye reduction after 24, 48 and 72 h growth on the different samples (normalized to the dye reduction of the growth control, which were uncoated glass substrates) is shown in Figure 6.

The dye reduction data shows that none of the tested samples were toxic to the GM-K cells. The maximum dye reduction for both **SMAMP@Au_Si** and **SMAMP@Au_PSB@Si** was obtained at 500 nm spacing (Figure 6a,b, respectively). Optical microscopy images (Figure S3) showed no significant differences between the morphology of keratinocytes grown on either the growth control, **SMAMP@Au_Si** or **SMAMP@Au_PSB@Si** surfaces. This was further confirmed with fluorescence microscopy, as most of the cells grown on the tested surfaces were viable (green) and only few keratinocytes were found membrane-compromised (red, Figure S4). The dye reduction data for **SMAMP@PSB** surfaces showed no significant difference for the different spacings tested (Figure 6c). The optical microscopy and the live-dead assay images of these µCP samples can be found in our previous published work [47]. Overall, the cell metabolism on the structured surfaces was higher than on the unstructured control surfaces (Figure 6d).

## 3. Discussion

Two sets of bifunctional structured surfaces with different spacing and stiffness were prepared by colloidal lithography (CL) and microcontact printing (µCP). The CL surfaces (spacing: 200 nm–2 µm) were thin polymer layers, while the µCP surfaces (spacing: 1–8.5 µm) were thicker polymer networks. QNM-AFM studies showed that the different polymer architectures used (thin layers vs. networks) led to different local moduli: the CL surfaces had much higher moduli (DMT modulus: 20 ± 0.8 GPa) than the µCP surfaces (DMT modulus: 42 ± 1.1 MPa). It was further observed that the different surface architectures led to different in the surface bioactivities despite the fact that the materials were made from similar bioactive polymers.

From the protein adhesion studies, it was found that the CL surfaces with smaller spacings (200–500 nm) were fully protein repellent, i.e., protein adhesion was below the detection limit of the SPR (< 0.1 ng mm^−2^). However, a small amount of protein consistently adhered to the surfaces with the larger spacings (1–2 µm, 0.2 and 0.5 ng mm^−2^, respectively). At these bigger spacings, not only the **PSB** patches, but also the cationic protein-adhesive **SMAMP** patches were larger. These could interact electrostatically with the overall negatively charged protein molecules and adsorbed protein. On the other hand, all µCP surfaces were fully protein repellent, regardless the spacing of the printed **SMAMP** structures, which were two to three times larger then the CL structures. This could be related to the lower local elastic modulus of the µCP materials at the air-polymer interface. Alternatively (or additionally), the swellability of these networks could be so much higher compared to the thin layers obtained by CL that this effect also reduced protein adhesion. Note that the moduli were measured on the dry samples; a higher swellability would in turn lead to even lower moduli for the µCP samples in aqueous media. Furthermore, the µCP surfaces should have many freely dangling chain ends at the polymer-liquid interface, resulting in a less sharp interface when immersed in aqueous medium. These chains could entropically shield the interface from protein adsorption, even though they consist of protein-adhesive **SMAMP** patches [47]. There will be a competition between this entropic effect and the adhesion enthalpy gained by the protein-**SMAMP** interaction. As it was found that proteins do not adhere, the Gibbs free energy of the protein adhesion process on µCP surfaces should be positive and thus unfavorable [47]. The entropic shielding theory has been previously discussed in the literature [47,54,55]. The surface protection from protein adsorption by entropic shielding is only possible when the surfaces are functionalized with polymer networks that can swell in the protein medium [54,55]. Thus, if the CL polymer layers swell less, this in turn reduces the entropic effect, leading to a lower protein repellency compared to the µCP materials.

The results from the antimicrobial activity assay of the CL surfaces against *E*. *coli* (length 2–4.5 μm; diameter 0.8–2.3 μm [47]) show that the highest activity was obtained when the spacing was 500 nm (4% CFUs) or 1 µm (0% CFUs). The smallest spacing (200 nm) and the biggest spacing (2 µm) had a lower activity (25% CFUs and 17% CFUs, respectively). At the smallest spacing of 200 nm, the **SMAMP** patches were probably too small compared to the *E*. *coli* cell size, leading to an insufficient interaction between the **SMAMP** and the bacterial cells. Hence, the antimicrobial activity was decreased. The decreased antimicrobial activity at the 2 µm spacing was surprising at the first glance, considering that bigger **SMAMP** patches should provide enough contact area with the bacteria and thus killed them more efficiently. However, with the **SMAMP** patches, the **PSB** domains also increase, and the activity of those domains is very low, as found for the homogenous control samples. Overall, the bifunctional CL surfaces had a lower antimicrobial activity than the monofuntional surfaces (i.e., **SMAMP@Au_Si**). This could be because the **PSB** regions shield the **SMAMP** regions and thus reduce their bioavailability.

For the bifunctional µCP materials, quantitative or near-quantitative killing was observed for all spacings. Here, the surface architecture (thin CL layer vs. thick µCP network) played a significant role. Apparently, the softer µCP materials could interact more efficiently with the bacteria than the CL samples. It is possible that the deformability of the µCP samples enabled a higher contact area per bacterium than the CL samples, so that the bacterial membranes were more efficiently destabilized by the µCP materials. Both the µCP and CL surface are hydrophilic materials. Previous reports by other groups indicate that softer hydrophilic samples adhere a lower amount of bacteria [37]. Even though the systems investigated in these studies had significantly lower moduli than the CL materials here presented, the same trends in antimicrobial activity and protein adhesion were observed for our materials: the softer materials were overall more efficient in eradicating bacteria, and adsorbed less protein.

Judging from the protein adhesion studies and the antimicrobial activity assay of this data set, the softer 3D µCP surfaces were the better option for obtaining materials with simultaneous antimicrobial activity and protein repellency. All µCP spacings (1–8.5 µm) were simultaneously protein repellent against fibrinogen and antimicrobially active against *E*. *coli*. On the other hand, it was difficult to find an optimal spacing for the stiffer 2D CL surfaces. In our previous work where the position of the **SMAMP** and **PSB** was reversed (i.e., **PSB@Au_SMAMP@Si**), the optimum spacing was 1 µm (0% CFUs and 0.1 ng mm^−2^), and the second best option was 500 nm spacing (5% CFUs and 0 ng mm^−2^) [46]. These data show that it was not possible to obtain complete simultaneous antimicrobial activity and protein repellency for the CL materials, possibly because these materials were thin, poorly swellable layers. Furthermore, the data also clearly shows that above 1 µm, the antimicrobial activity of the bifunctional CL surfaces became seriously compromised. There also seems to be a trend of higher protein adhesion with increasing spacing. Thus, the upper limit for optimal dual antimicrobial activity and protein repellency is clearly reached at a spacing of about 1 µm for the CL materials, whereas the µCP materials still have full protein repellency and a respectable, though not quantitative antimicrobial activity even at a spacing of 8.5 µm. These bioactivity differences are a result of differences in the surface architecture, which in turn led to different local swellability and elastic moduli.

The cell compatibility tests show that all fabricated materials were compatible with human gingival mucosal keratinocytes. Another significant result that can be derived from the data is that the cells had a higher metabolic activity on the structured CL and µCP surfaces compared to the unstructured control surfaces. As for the effect of the surface spacings on the cell viability, there was no clear trend observed: the metabolic activity on the 500 nm CL surfaces was the highest, while there was no significant difference between the differently spaced µCP surfaces. This is in line with the previously published reviews about cell-surface topology interaction: so far, there are no general rules that could explain cell behavior on structured surfaces [56,57]. Nevertheless, although the surface topology effects on cell adhesion and growth remain unclear, the presence of the topology itself would increase the surface area, which in the end should improve cell adhesion and growth, as observed in our data set. The effect of surface stiffness on cell growth is also not clear. For example, the 2 µm CL materials had a higher cell metabolism than the 2 µm µCP materials. On the other hand, the cells grown on the 1 µm CL materials were about as active as those grown on the 1 µm µCP materials. In a recent study by Gupta et al. [58], it was reported that keratinocyte proliferation increased with an increased PDMS surface stiffness (0.05–1.6 MPa) [58]. This could be an explanation as to why some CL surfaces, which were stiffer, had >150% of dye reduction, while the dye reduction of all the softer µCP surfaces was <150%.

## 4. Experimental

### 4.1. Surface Functionalization and Structuring by Colloidal Lithography

The target system is shown in Figure 1. Figure 1a shows the chemical structure of the polymers used, a SMAMP carrying *tert*-butyloxycarbonyl (Boc) protective groups (**Boc-SMAMP**) and the polyzwitterion **PSB**. These were synthesized as reported previously [46]. Figure 1b illustrates the surface structuring process, which also has been reported previously [46,48]. For the here presented material, a colloidal monolayer made from 2 μm polystyrene beads (obtained from Micromod GmbH, Rostock, Germany) was formed on a silicon wafer piece (1.5 × 1.5 cm^2^). This layer served as a lithographic mask for the subsequent evaporation of 5 nm chromium and 40 nm gold. After that, the colloidal mask was lifted off, yielding a gold-silicon contrast on the surface, which was functionalized site-selectively with UV-reactive linker molecules. The gold islands were functionalized with lipoic acid disulfide benzophenone (**LS-BP**, Figure 1b) by immersing the substrates into 5 mmol mL^−1^ **LS-BP** in toluene for 24 h. The substrates were then rinsed with toluene. After that, **boc-SMAMP** solution (10 mg mL^−1^ in dichloromethane (DCM); M_n_ = 100,000 g mol^−1^) was spin-coated onto the surfaces. The substrates were then UV-irradiated (λ = 254 nm; irradiation energy = 3 J cm^−2^) to covalently attach the polymer to the surface. Unbound polymer chains were removed by washing the substrates with DCM. Afterwards, a 20 mg mL^−1^ solution of triethoxy benzophenone silane (**3EBP**, Figure 1b) in toluene was spin-coated onto these substrates. It was heated for 30 min at 120 °C to selectively functionalize the silicon patches. After rinsing with toluene, a solution of **PSB** (M_n_ = 50,000 g mol^−1^) in trifluoroethanol (TFE) was spin-coated onto the surfaces, and they were further UV-irradiated (λ = 254 nm; irradiation energy = 3 J cm^−2^) to surface-attach the **PSB** chains. The unbound polymer chains were removed by washing the substrates with TFE. The surfaces were then immersed into 4 M HCl in dioxane for 15 h to remove the Boc protecting groups of **Boc-SMAMP** and generate **SMAMP** with primary ammonium groups. After that, the substrates were rinsed with ethanol and dried under nitrogen flow.

### 4.2. Surface Functionalization and Structuring by Microcontact Printing

Silicon substrates were functionalized and structured as illustrated in Figure 1c. First, silicon wafer pieces (1.5 × 1.5 cm^2^) were functionalized with the UV-active benzophenone crosslinker **3EBP** (20 mg mL^−1^ in toluene) by spincoating (3000 rpm, 1000 rpm s^−1^, 30 s). The substrates were then cured at 120 ^o^C for 30 min on a preheated hotplate, and washed with toluene. Afterwards, a **BP-COU-PSB** layer was spin-coated on top of the benzophenone functionalized surfaces (10 mg mL^−1^ **BP-COU-PSB** in TFE; 3000 rpm, 1000 rpm s^−1^, 10 s) and UV irradiated at λ = 254 nm (irradiation energy = 3 J cm^−2^) to form a surface-attached **PSB** network. The **BP-COU-PSB** used had 85% **PSB** repeat units, 5 mol% benzophenone repeat units, and 10 mol% coumarin repeat units as described in [47]. Any loosely attached polymer chains were removed by rinsing with TFE. The µCP process was conducted as previously reported [47,49]. **Boc-NBD-SMAMP** containing 10 mol% nitrobenzoxadiazole as described in [47] was used as ink for microcontact printing. This ink was applied in its Boc protected form to obtain proper wetting of the PDMS stamp (stamp structure: parabolic; stamp spacing: 1 µm, 2 µm, or 8.5 µm). The stamp had been prepared as reported previously [47]. Surface structuring with 1 µm and 2 µm spacing stamps was performed as follows: **Boc-NBD-SMAMP** solution was spin-coated onto a glass slide (ink concentration: 5 mg mL^−1^ for 1 µm spacing stamp, 2 mg mL^−1^ for 2 µm spacing stamp; the solvent was a mixture of 30% *v*/*v* DCM and 70% *v*/*v* toluene; spin coating parameters: 150 rpm, 150 rpm s^−1^, 5 s). The stamp was then placed on the glass slide to transfer the **Boc-NBD-SMAMP** ink from the glass slide to the stamp. The **Boc-NBD-SMAMP** loaded stamp was then brought onto conformal contact with the **PSB network** surface (printing force 30 N, printing time 5 s). For the stamp with 8.5 µm spacing, one drop of **Boc-NBD-SMAMP** solution (3 mg mL^−1^ in THF) was pipetted onto the stamp, and the solvent was allowed to evaporate. Afterwards, the stamp was brought onto conformal contact with the **PSB network** surface (printing force 15 N, printing time 20 s). The printed **Boc-NBD-SMAMP** was then UV-irradiated (λ = 254 nm; irradiation energy = 3 J cm^−2^). Any loosely attached polymer chains were then removed by washing the substrates with toluene. The Boc protecting groups of **Boc-NBD-SMAMP** were removed by immersing the substrates in HCl solution (4 M in dioxane; 15 h), so that **SMAMP@PSB** is obtained. Finally, the substrates were washed with ethanol and dried under nitrogen flow.

### 4.3. Preparation of Reference Surfaces

Four control surfaces (**SMAMP monolayer**, **SMAMP network**, **PSB monolayer**, and **PSB network**) were prepared as previously reported [46,47]. First, silicon substrates were functionalized with 20 mg mL^−1^ solution of **3EBP** in toluene, followed by heating for 30 min at 120 °C. After rinsing with toluene, the respective polymer solution was spin coated onto the benzophenone-functionalized substrates as described below.

To obtain the **SMAMP monolayer**, a solution of **Boc-SMAMP** (M_n_ = 100,000 g mol^−1^, 10 mg mL^−1^ in dichloromethane (DCM)) was used; for the **SMAMP network**, a **BP-NBD-SMAMP** copolymer consisting of 80 mol% **Boc-SMAMP** repeat units, 10 mol% benzophenone repeat units, and 10 mol% nitrobenzoxadiazole repeat units (M_n_ = 100,000 g mol^−1^, 10 mg mL^−1^ in a mixture of 30% *v*/*v* DCM-70% *v*/*v* toluene) was used [47]. The polymers were then UV-irradiated (λ = 254 nm; irradiation energy = 3 J cm^−2^), washed with toluene, and deprotected with 4 M HCl in dioxane.

The **PSB** reference samples were prepared from either **PSB** or **BP-COU-PSB** (M_n_ = 50,000 g mol^−1^ each, 10 mg mL^−1^ in trifluoroethanol (TFE)). **BP-COU-PSB** solution (copolymer of 85 mol% PSB, 5 mol% benzophenone, and 10 mol% coumarin) was used to generate the **PSB network**. **PSB** solution was used to prepare the **PSB monolayer** (spin coater: 3000 rpm, 1000 rpm s^−1^, 30 s). After that, the substrates were UV-irradiated (λ = 254 nm; irradiation energy = 3 J cm^−2^). The unreacted polymer was removed by immersion into stirred TFE for 18 h under ambient conditions. The samples were then dried under nitrogen flow.

### 4.4. Surface Characterization

Most physical characterization methods and techniques were performed as reported previously [46,47], and brief details are described below. The quantiatiative nanomechanical measurement is described in more fully.

#### 4.4.1. Atomic Force Microscopy

Surface topology images were recorded on a Dimension Icon AFM from Bruker (Karlsruhe, Germany). Bruker ScanAsyst Air cantilevers (length: 115 µm; width: 25 µm; spring constant: 0.4 Nm^−1^; resonance frequency: 70 kHz) and OTESPA-R3 cantilevers (length: 160 µm; width: 40 µm; spring constant: 26 Nm^−1^; resonance frequency: 300 kHz) were used. Quantitative Nanomechanical (PeakForce-QNM) measurements were performed using the same device. Bruker Tap-525 cantilevers (length: 125 µm; width: 40 µm; spring constant: 200 Nm^−1^; resonance frequency: 525 kHz) were used for the surfaces patterned with colloidal lithography (CL), and Bruker SNL-A cantilevers (length: 120 µm; width: 25 µm; spring constant: 0.427 Nm^−1^; resonance frequency: 65 kHz) were used for the surfaces patterned by microcontact printing (µCP). Three parameters were calibrated prior the measurements: deflection sensitivity, spring constant, and tip radius. The deflection sensitivity was calibrated using a sapphire standard sample (Bruker, PFQNM-SMPKIT-12M, SAPPHIRE-12M), and the results showed that Tap-525 had a deflection sensitivity of 74.88 nm V^−1^, and SNL-A had a deflection sensitivity of 51.52 nm V^−1^. The spring constant was calibrated using thermal tune, which is only valid for spring constant smaller than 1 Nm^−1^. Therefore, only SNL-A was calibrated (spring constant: 0.427 Nm^−1^). For TAP-525, the value of 200 Nm^−1^ (given by the manufacturer) was used. The tip radius was calibrated using an absolute method. For this, a titanium carbide standard sample (Bruker, PFQNM-SMPKIT-12M, RS-12M) was used to perform a Tip Check. Then, the titanium carbide standard sample was replaced by the unkown samples (i.e., the bifunctional structured surfaces). The value of the PeakForce (PF) setpoint was then manually adjusted to obtain a 2-5 nm surface deformation (usually the PF setpoint was in the range of 5.6–6 nN for Tap-525 (used for structured surfaces obtained by CL) and of 4.5–5.4 nN for SNL-A (for structured surfaces obtained by µCP)). Afterwards, the curve of Force (nN) vs. z position (nm) was captured and the indentation depth was measured using the Nanoscope Analysis 1.5 software. The obtained indentation depth value was inserted into the Tip Check image of the titanium carbide standard sample to calculate the tip radius. The results showed that the tip radius of the Tap-525 was 17.33 nm, and that of the SNL-A was 11.57 nm. The obtained topology and modulus images were processed with Nanoscope Analysis 1.5 software. The height profiles were analyzed and processed with Gwyddion 2.47 software.

#### 4.4.2. Contact Angle Measurements

The static, advancing, and receding contact angles were measured by using OCA 20 set-up (Data Physics GmbH, Filderstadt, Germany) at five different positions on each sample, and the average values were calculated.

#### 4.4.3. Surface Plasmon Resonance Spectroscopy

Surface plasmon resonance spectroscopy (SPR) was used to study the protein adhesion on the surfaces, which was performed in the kinetics mode on an RT2005 RES-TEC device (Res-Tec, Framersheim, Germany) in Kretschmann configuration. SPR substrates were homemade (LaSFN9 glass from Hellma GmbH, Müllheim, Germany; coated with 1 nm Cr and 50 nm Au at the Clean Room Service-Center (RSC) of the Department of Microsystems Engineering, University of Freiburg, using the device CS 730 S, Von Ardenne, Dresden, Germany). These substrates were used for the µCP samples. For the CL samples, the colloidal monolayer was deposited on SPR substrates using the same procedure as for the silicon wafers described above. Then, a thin layer of chromium (5 nm, adhesive layer) was evaporated. After that, SiO_2_ was sputtered through the lithographic mask (40 nm) at the Fraunhofer IAF using a Cluster-Line 200 II DC-pulsed sputter by EVA tech (750W, at room temperature, source material SiO_2_). The colloids were removed with scotch tape, and the samples were washed and dried under nitrogen flow.

In addition to the SPR kinetics measurements, full reflectivity angular scans (reflectivity vs. measurement angle) were recorded before and after each run, and the thickness of each sample was simulated using the Fresnel equations using the ‘Winspall’ software (Version 3.02, Res-Tec, Framersheim, Germany).

### 4.5. Biological Assays

#### 4.5.1. Antimicrobial Activity Assay

A modified version of the Japanese Industrial Standard JIS Z 2801:2000 (‘Antibacterial Products Test for Anti-bacterial Activity and Efficacy’) was used to analyze the antimicrobial activity of the fabricated surfaces, as reported previously [46,50]. Briefly, *E*. *coli* (ATCC25922) was cultured overnight in tryptic soy broth and diluted 1:10. After 3-4 h, 150 µL of the *E*. *coli* bacterial culture was mixed with 100 mL of 0.9% sterile NaCl solution in a chromatography spray bottle under continuous stirring. The tested samples (including negative and positive controls) were sprayed with the bacterial suspension, covered and incubated in a humid chamber (2–4 h, 37 °C, aerobic conditions, 5% CO_2_). Afterwards, 50 µL of sterile 0.9% NaCl solution was added onto the samples and incubated for 2 min. Thus the dispersed bacteria were aspirated with a pipette, and re-pipetted twice. With this procedure, a representative amount of bacteria was transferred from the surface into the NaCl solution.

The NaCl solution was then spread over Columbia blood agar plates. These agar plates were incubated overnight at 37 °C without any agitation. Each experiment was performed at least twice, where the number of colony forming units (CFUs) were counted with the software ‘Quantity One’. The growth percentage relative to the negative control (growth control) and the positive (dead) control was reported as CFU and calculated via the following Equation (2):

(2) $$\%\;\mathrm{Growth}\;=\;({\mathrm{CFUs}}_{\mathrm{sample}}\;-\;{\mathrm{CFUs}}_{\mathrm{positive}\;\mathrm{control}})/({\mathrm{CFUs}}_{\mathrm{negative}\;\mathrm{control}}\;-\;{\mathrm{CFUs}}_{\mathrm{positive}\;\mathrm{control}})\;\times \;100\%$$

#### 4.5.2. Alamar Blue Assay and Optical Microscopy

*Ethics Statement*: Gingival mucosal keratinocytes were obtained from human volunteers who previously signed their consent according to the Helsinki declaration. This was approved by the Ethics Board of the Albert-Ludwigs University Freiburg, Germany (ethics vote number 381/15).

As previously reported [48,50], gingival mucosal keratinocytes (GM-K), immortalized with HPV-16, were cultured in keratinocytes growth medium (KGM, Promocell, Heidelberg, Germany) with accompanying supplement at concentrations as supplied by the manufacturer: bovine pituitary extract: 0.004 mg mL^−1^; insulin: 5 μg mL^−1^; epidermal growth factor (EGF): 0.125 ng mL^−1^; hydrocortisone: 0.33 µg mL^−1^; epinephrine: 0.39 µg mL^−1^; transferrin: 10 μg mL^−1^; kanamycin: 100 μg mL^−1^; CaCl_2_: 0.06 mM. At a cell confluence of 70–90%, the cells were detached with accutase (Sigma-Aldrich, Munich, Germany) and re-suspended in supplement/antibiotic-free KGM. The cells were seeded out onto the samples (1 mL of cell dispersion with a concentration of 1.5 × 10^5^ cells mL^−1^ in supplement/antibiotic free medium). For 5h, the 12 well plates containing the cells were incubated at 37 °C/5% CO_2_ to allow adhesion and the settlement on the coverslips. Then, 500 µL of medium was carefully aspirated from the samples and replaced by 500 µL fresh medium containing double the normal supplement concentration. Mixed with the remainder of the cell medium, this yielding a normal supplement concentration. For time-dependent analytics, the cells were further cultivated for 18 more hours (total 24 h), 42 h (total 48 h) and 66 h (total 72 h), respectively. At each time point, positive (dead) and negative (growth) controls were generated. For the negative controls, 1 mL medium was removed and replaced by 1 mL of fresh medium. For the positive controls, 500 µL of the medium was aspirated, and 500 µl of 60% isopropanol and 500 µL of fresh medium were added, yielding a 30% isopropanol solution. Optical micrographs of the keratinocytes grown on all the tested surfaces and the growth control were recorded with a Leica DMIL microscope with a Leica D-LUX-3 CCD camera at 200x magnification. For the Alamar Blue assay, all the tested samples and controls (positive and negative) were cultivated for another 30 min. Afterwards, 110 µL Alamar Blue (AbD Serotec, Oxford, UK) was slowly pipetted into the wells, yielding a 10% solution. The wells were agitated gently to obtain a homogenous dispersion. After re-incubation of the cells for 2 h, the supernatant of each well was aspirated and collected in a 1.5 mL Eppendorf tube. They were then centrifuged at 1000× *g* for 5 min. After that, the fluorescence intensity of the supernatant was measured (excitation at 540 nm; measurement at 590 nm) on an Infinite 200 plate reader (Tecan, Männedorf, Switzerland). The data were analyzed according to the Alamar Blue manufacturer’s instructions.

#### 4.5.3. Live-Dead Staining of Keratinocytes Grown on Functionalized Structured Surfaces

After the cultivation of the keratinocytes for 72 h in the Alamar Blue assay, live-dead stainings were performed. Instantly after the removal of the supernatant, the GM-K cells were washed twice with PBS buffer. For cell staining, green fluorescent Syto16 nucleic acid stain (Molecular Probes, Eugene, OR, USA), which can permeate the membranes of all cells, was diluted 1:200 in keratinocyte growth medium (Promo Cell, Heidelberg, Germany) and added to the cells. For the “dead” cell staining, propidium iodide (Sigma-Aldrich GmbH, Steinheim, Germany; dilution 1:1000), which only permeates the membrane of damaged cells, was used. The GM-K cells were stained for 30 min at 37 °C in humidified air with 5% CO_2_, and then washed twice with PBS. Afterwards, images of the samples were taken with a Keyence BZ-9000E fluorescence microscope with the software BZ II Analyser and BZ II Viewer, Neu-Isenburg, Germany. Green fluorescence was excited at ca. 490 nm, red fluorescence at 536 nm. The image contrast and brightness were adjusted for better visualization.

## 5. Conclusions

This study presents a comparison of two types of bifunctional structured surfaces that were made from the antimicrobial polycation SMAMP and the protein repellent polyzwitterion PSB. The first type of materials was fabricated by a colloidal lithography (CL) based process, where thin bioactive polymer layers were immobilized onto pre-structured surfaces with a chemical contrast (spacing: 200 nm–2 µm) to obtain 2D structured surfaces. The second type of materials (spacing: 1 – 8.5 µm) was fabricated using a microcontact printing (µCP) based process, where 3D SMAMP patches were printed onto a PSB network. Thus, bifunctional bioactive structured surfaces with different spacings and architecture (2D monolayers vs. 3D networks, respectively) were fabricated. These architectures led to different local elastic moduli at the polymer-air interface: the 2D surfaces were much stiffer (DMT modulus = 20 ± 0.8 GPa) than the 3D surfaces (DMT modulus = 42 ± 1.1 MPa). The effects of the surface topology and stiffness on antimicrobial activity against *E*. *coli*, protein repellency against fibrinogen, and cell compatibility with human gingival mucosal keratinocytes were investigated. The data sets show that the softer 3D µCP surfaces made from polymer networks had simultaneous antimicrobial activity, protein repellency, and cell compatibility at all spacings. As for the stiffer 2D CL surfaces, a reduced bioactivity performance was observed, and quantitative simultaneous antimicrobial activity and protein repellency was not obtained. However, the cell compatibility could be maintained at all spacings. The optimum spacing for the CL surfaces was in the range of 500 nm–1 µm. Thus, from the overall data set obtained in this study, it can be concluded that the bioactivity of the soft polymer network could be more easily optimized than that of the stiff CL surfaces. Furthermore, the soft 3D polymer networks had a broader spacing range of optimal or near-optimal bioactivity. Hence, this soft material is a promising candidate for applications in biomedical field, e.g., for implants and tissue engineering. However, further studies are needed before these materials can be put into real-life applications. For example, their fabrication process needs to be simplified and scaled to larger surface areas, and their stability under application conditions needs to be verified.

## Figures and Tables

**Table 1:** Average amount of adhered fibrinogen (in ng mm^−2^) on the different materials studied. Part of the data was previously reported [46,47].

Sample	Adhered Fibrinogen (ng mm^−2^)	Elastic Modulus (MPa)
**PSB monolayer**	11	n.a
**SMAMP monolayer** **PSB network** **SMAMP network**	13 0 6.6	n.a 236 ± 18 58 ± 0.4
**CL materials: SMAMP@SiO_2__Au**		
200 nm	10.8	n.a
500 nm	2.8	n.a
1 µm	16.3	(26 ± 0.7) × 10^3^
2 µm	7.0	n.a
**CL materials: SMAMP@SiO_2__PSB@Au**		
200 nm	0.0	n.a
500 nm	0.0	n.a
1 µm	0.2	(20 ± 0.8) × 10^3^
2 µm	0.5	n.a
**µCP materials: SMAMP@PSB**		
1 µm	0.0	n.a
2 µm	0.0	42 ± 1.1
8.5 µm	0.0	n.a

## Supplementary Materials

The following are available online. Table S1: Contact angle data (static, advancing and receding contact angles) of surfaces with 200 nm to 2 µm spacing; Figure S1: Height images (AFM) of polystyrene colloid monolayers with 2 µm diameter; Figure S2: Reflectivity curves after each processing step of surfaces with 2 µm spacing by surface plasmon resonance spectroscopy (SPR); Table S2. Average layer thickness for the 2 µm patterned functionalized surfaces after every processing step; Figure S3: Optical micrographs of human keratinocytes (GM-K) grown on surfaces 200 nm to 2 µm spacing; Figure S4: Live-dead staining images of human keratinocytes (GM-K) grown on surfaces 200 nm to 2 µm spacing.
