# Supplementary material for: Bifunctional Bioactive Polymer Surfaces with Micrometer and Submicrometer-Sized Structure: The Effects of Structure Spacing and Elastic Modulus on Bioactivity
Source: Molecules. 2019 Sep 16;24(18):3371. doi: 10.3390/molecules24183371 (PMC6767307; doi:10.3390/molecules24183371)
Supplement: Supplementary file 1 [file molecules-24-03371-s001.pdf]

Bifunctional Bioactive Polymer Surfaces with Micrometer and Submicrometer-sized Structure: The Effects of Structure Spacing and Elastic Modulus on Bioactivity

Sarah M. Elsayed [1, 2, §], Vania Tanda Widyaya [1,§], Yasir Shafi [1], Alice Eickenscheidt [1], and Karen Lienkamp [1,*]

[1] Freiburg Center for Interactive Materials and Bioinspired Technologies (FIT) and Department of Microsystems Engineering (IMTEK), Albert-Ludwigs-Universität, Georges-Köhler-Allee 105, 79110 Freiburg, Germany;
[2] Department of Advanced Technology and New Materials Research Institute, City of Scientific Research and Technology Applications, New Borg El-Arab City, 21934 Alexandria, Egypt;
sarah.mahmoud@imtek.uni-freiburg.de (S.M.E.); vania.widyaya@imtek.uni-freiburg.de (V.T.W.); myaasir@outlook.com (Y.S); alice.eickenscheidt@imtek.uni-freiburg.de (A.E.).
* Correspondence: lienkamp@imtek.uni-freiburg.de; Tel.: +49-761-203-95090.
§ contributed equally

Supporting Information

1. Contact angle results

Table S1. Contact angle data (static, advancing and receding contact angles) for the structured functionalized surfaces **SMAMP@Au_Si** and **SMAMP@Au_PSB@Si**. Contact angles of Si, the polymer monolayers, and 200 nm, 500 nm, 1 µm and 2 µm spacing structured surfaces have been previously published [43] and presented here for comparison.

**Sample type**	**Contact angle (°)**		
	θ_{static}	$\theta_{advancing}$	$\theta_{receding}$
**Si**	71 ± 1	75 ± 3	41 ± 3
**PSB monolayer**	34 ± 3	35 ± 3	22 ± 1
**SMAMP monolayer**	59 ± 3	61 ± 3	33 ± 3
**SMAMP@Au_Si**			
200 nm	56 ± 3	55 ± 3	33 ± 2
500 nm	60 ± 2	69 ± 2	45 ± 1
1 µm	63 ± 3	71 ± 4	47 ± 3
2 µm	54 ± 3	66 ± 3	48 ±2
**SMAMP@Au_PSB@Si**			
200 nm	52 ± 3	56 ± 3	21 ± 1
500 nm	56 ± 2	57 ± 1	27 ± 2
1 µm	53 ± 3	59 ± 2	34 ± 3
2 µm	39 ± 2	40± 0	36± 0

2. Atomic force microscopy

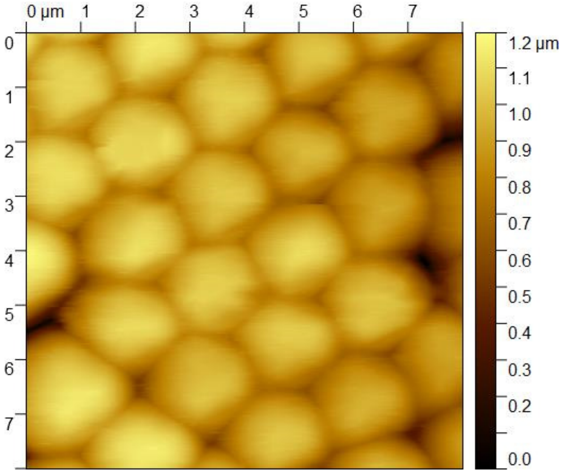

Figure S1: Atomic force microscopy (AFM) height images of polystyrene colloid monolayers with 2 µm diameter.

3. Surface Plasmon Resonance Spectroscopy

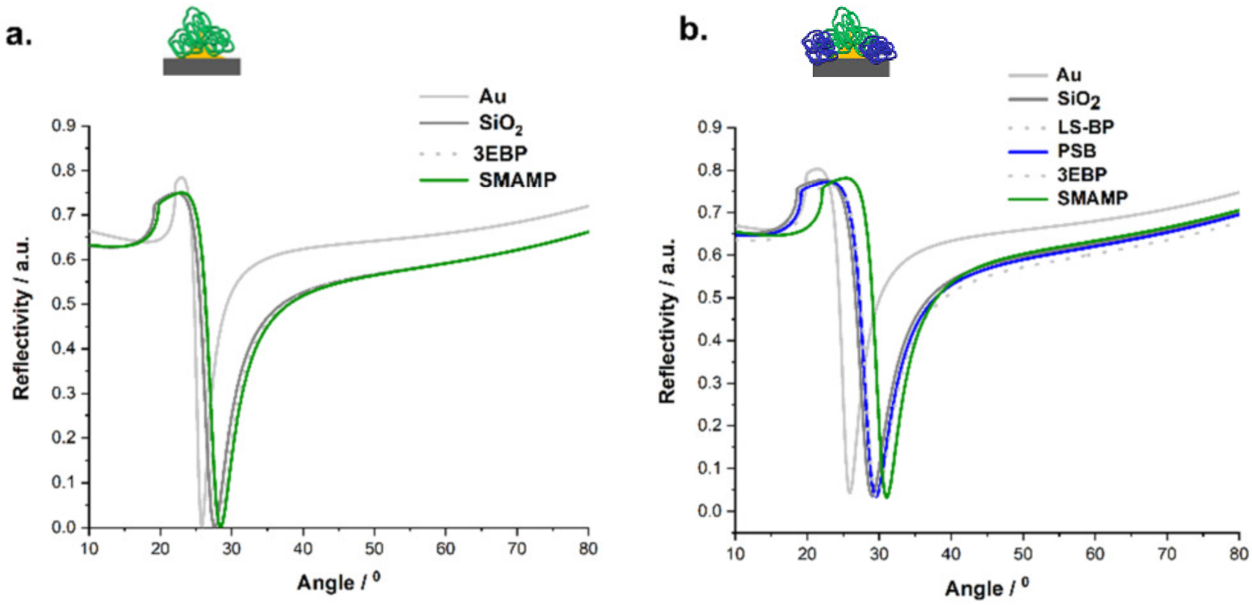

Figure S2. Reflectivity curves after each processing step of the 2 µm functionalized surfaces studied by surface plasmon resonance spectroscopy (SPR) for a. **SMAMP@SiO₂_Au**; b. **SMAMP@SiO₂_PSB@Au**.

Table S2. Average layer thickness and permittivity (ε' = real part, ε'' = imaginary part) for the 2 μm patterned functionalized surfaces calculated from fits to the surface plasmon resonance (SPR) curves

	**SMAMP@SiO2_PSB@Au**			**SMAMP@SiO2_ Au**		
	Layer thickness/nm	ε'	ε''	Layer thickness/nm	ε'	ε''
LaSNFN9 glass		3.4036	0		3.5736	0
Cr	0.40	-6.423	20	0.6	-6.263	20
Au	41.0	-11.85	1.3	50.3	-11.462	1.68
SiO2	10.9	2.13	0	10.5	2.13	0
LS-BP	2.0	2.25	0	0	2.25	0
PSB	9.0	2.04	0	0	2.04	0
3EBP	0.9	2.25	0	2.0	2.25	0
SMAMP	10.0	2.08	0	10.0	2.08	0

4. Optical micrographs of human keratinocytes

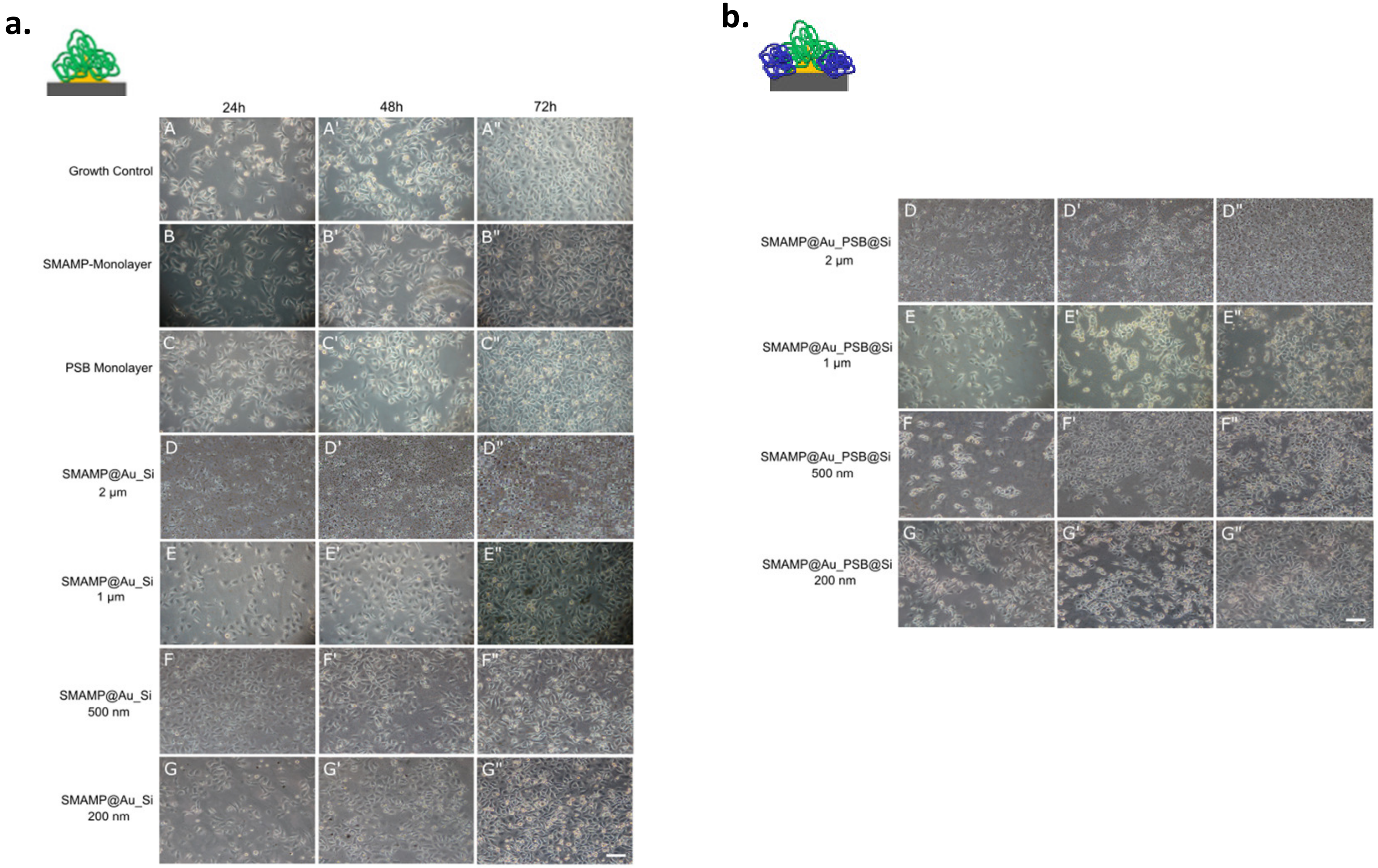

Figure S3. Optical micrographs of human keratinocytes (GM-K) after 24 h (A-G), 48 h (A′-G′) and 72 h (A′-G′′) growth on a. **SMAMP@Au_Si** and b. **SMAMP@Au_PSB@Si** functionalized surfaces with 2 µm ,1 µm , 500 nm and 200 nm spacing. Scale bars: 100 µm.

5. Live-Dead Staining of Keratinocytes

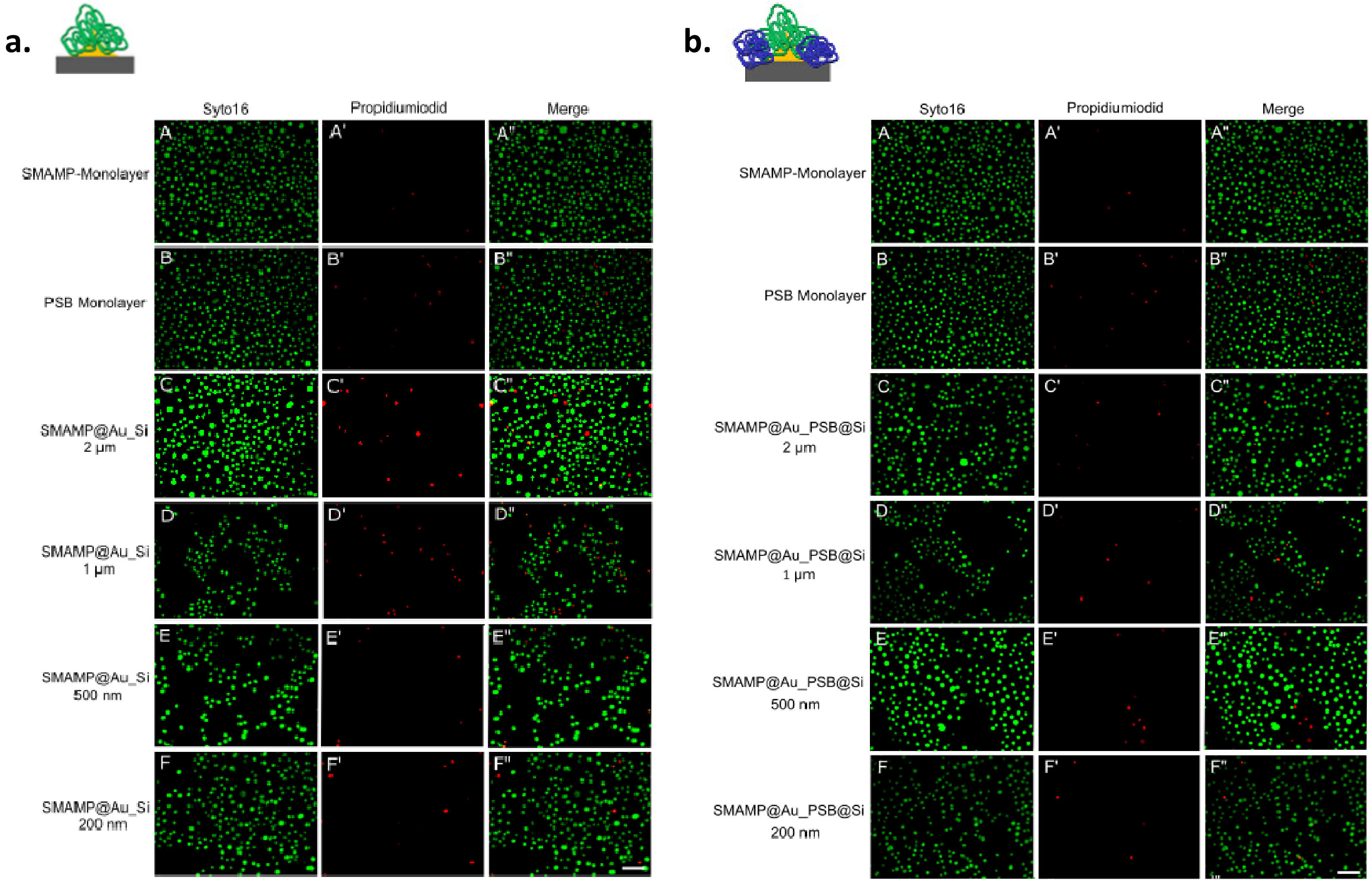

Figure S4 . Live- dead staining images of human Keratinocytes (GM-K) after 72 h grown on a. **SMAMP@Au_Si** and b. **SMAMP@Au_PSB@Si** functionalized surfaces with 2 µm, 1 µm, 500 nm and 200 nm spacing. The green stain (SYTO 16, A-F) visualizes live cells and the red stain (propidium iodide, A′-F′) the dead cells. Merged images are an overlay of both (A″-F″). Scale bars: 100 µm.
